# Evaluation of reference genes at different developmental stages for quantitative real-time PCR in *Aedes aegypti*

**DOI:** 10.1038/srep43618

**Published:** 2017-03-16

**Authors:** Najat Dzaki, Karima N. Ramli, Azali Azlan, Intan H. Ishak, Ghows Azzam

**Affiliations:** 1 School of Biological Sciences, Universiti Sains Malaysia, 11800 Penang, Malaysia; 2 Vector Control and Research Unit, School of Biological Sciences, Universiti Sains Malaysia, 11800 Penang, Malaysia

## Abstract

The mosquito *Aedes aegypti (Ae. aegypti*) is the most notorious vector of illness-causing viruses such as Dengue, Chikugunya, and Zika. Although numerous genetic expression studies utilizing quantitative real-time PCR (qPCR) have been conducted with regards to *Ae. aegypti*, a panel of genes to be used suitably as references for the purpose of expression-level normalization within this epidemiologically important insect is presently lacking. Here, the usability of seven widely-utilized reference genes i.e. actin (*ACT*), eukaryotic elongation factor 1 alpha (*eEF1α*), alpha tubulin (*α*-*tubulin*), ribosomal proteins L8, L32 and S17 (*RPL8, RPL32* and *RPS17*), and glyceraldeyde 3-phosphate dehydrogenase (*GAPDH*) were investigated. Expression patterns of the reference genes were observed in sixteen pre-determined developmental stages and in cell culture. Gene stability was inferred from qPCR data through three freely available algorithms i.e. BestKeeper, geNorm, and NormFinder. The consensus rankings generated from stability values provided by these programs suggest a combination of at least two genes for normalization. *ACT* and *RPS17* are the most dependably expressed reference genes and therefore, we propose an *ACT/RPS17* combination for normalization in all *Ae. aegypti* derived samples. *GAPDH* performed least desirably, and is thus not a recommended reference gene. This study emphasizes the importance of validating reference genes in *Ae. aegypti* for qPCR based research.

*Aedes aegypti(Ae. aegypti*) is widely regarded as the primary vector of arthropod-borne viruses (arboviruses) such as Dengue, Zika and Chikugunya. The past year has seen the mosquito garnering much international attention due to the roles it may have played in the widespread propagation of Zika, although historically the first-reported association of the virus to *Ae. aegypti* dates as far back as 50 years ago^1^. This reputation as a host to such organisms has created an *Ae. aegypti* research base more revolved around the viruses and parasites it carries, rather than the insect itself. However, its relatively recent re-emergence in parts of Europe and North America as well as associations with an ever-growing list of zoonotic diseases have renewed interests in the species as its own organism^2^,^3^. Studies involving gene expression and regulation are therefore imminent, as they will not only elucidate the biological significance of any particular gene within *Ae. aegypti*, but additionally provide a clearer understanding of the complexities behind the networks within which host-virus interaction occurs.

Presently, quantitative real-time polymerase chain reaction (qPCR) remains the most accessible and widely-applied technique for such purposes. The method not only requires minimal nucleic acid quantities when compared to more traditional quantification assays such as northern-blot, but is faster and more reproducible^4^. The ready availability of reagents and automated platforms has also added to its burgeoning popularity^5^. Despite its advantages, interpretation of qPCR data is made difficult due to inconsistencies in protocol as well as in template quality and enzymatic efficiencies^6^,^7^. Normalization of data against a ‘housekeeping’ or reference gene is therefore critical, as it compensates for differences in starting cDNA quantities amongst samples caused by variations encountered along the RNA extraction and subsequent reverse transcription steps^8^,^9^. It involves comparing the ratios of expression levels of the target gene against that of the selected reference gene(s)^10^. *ACT* and *GAPDH* are examples of genes utilized heavily for this purpose. However, it has quickly emerged that no one gene is stably expressed under all developmental and experimental conditions^11^,^12^,^13^,^14^. Algorithms such as BestKeeper, geNorm, and NormFinder have been developed to identify the best-fit reference gene to use with consideration to one’s protocol and biological samples^10^,^15^. Nonetheless, choosing a set of genes as reference rather than just one may in fact be necessary to normalize gene expression experiments^16^,^17^,^18^.

Up to date, the most commonly used gene for normalization in RNA quantification methods in *Ae. aegypti* is the *RibosomalProteinS17 (RPS17*)^19^,^20^,^21^,^22^,^23^. However, application of a singular reference gene for all tissue and morphological types of the developing mosquitos an arguably flawed scientific approach. This is especially true for *Ae. aegypti* as in addition to undergoing complete metamorphosis, the insect spends half its life-cycle as an aquatic organism. This indicates that it is exposed to many variables throughout its lifetime; not only in terms of natural progression, but from additional environmental influences. Growth and development patterns of larvae and adult mosquitoes have indeed been shown to be greatly affected by several factors such as diet and temperature^24^,^25^,^26^. There is thus a need for the stability of reference genes at different points of development to be validated to ensure robustness in gene expression normalization where samples are from individuals of a specific developmental stage.

Here, we comprehensively evaluated seven candidate reference genes i.e. *ACT, eEF1α, GAPDH, α*-*tubulin, RPL8, RPL32* and *RPS17* at nine points of development as well as inAag2cells. Three programs, namely geNorm, BestKeeper, and NormFinder, were used to analyse their stability and to rank the reference genes for usability at any particular point of development. We also applied our findings to the normalization of a chosen target gene i.e. CTP synthase (*CTPsyn*) to validate the consensus rankings generated. The suitable reference gene(s) can be applied for normalization of qPCR data for whole organism *Ae. aegypti* tissue at multiple developmental stages as well as cell culture.

## Results and Discussion

### Primer evaluation and amplification efficiency of candidate reference genes

Seven candidate genes from three functional classes were investigated: (i) structure-related genes: *ACT* and *α*-*tubulin*, (ii) ribosomal and protein-production genes: *eEF1a, RPS17, RPL8* and *RPL32*, and (iii) metabolism-related gene: *GAPDH*. Primer pairs were evaluated through standard curve generation with serially diluted pooled cDNA. Efficiency(E) and linear regression coefficient (R^2^) values are observed to determine the performance of the designed primers in detecting and amplifying cDNA at very high to very low concentrations. All recorded acceptable E values between 92.5 and 100.8% with R^2^ values ranging from 0.979 to 0.997 (Table 1). Amplification specificity was displayed through the production of a singular peak in melt-curve analysis. Purified qPCR products were sequenced to show specificity and accuracy whereby (a) each primer produced a singular sequence output, and (b) the sequence aligns with the cDNA of the expected gene through BLAST (https://blast.ncbi.nlm.nih.gov/Blast.cgi). Sequences are available on GenBank’s BankIt depository with the accession numbers KY000701 to KY000707. Post-run 2% agarose gel further confirmed single amplicon production (see Supplementary Figure S1B).

### Expression levels and sample integrity

**Figure 1:**
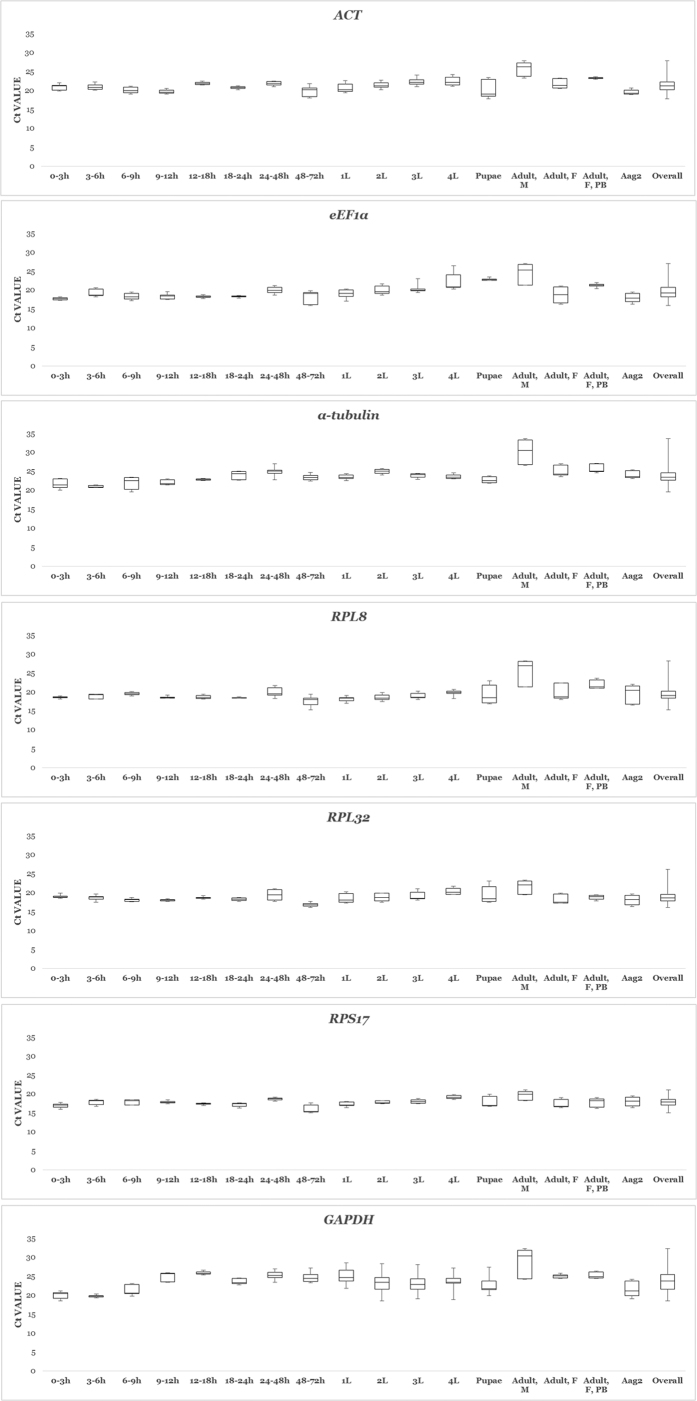
Box-whisker plots depicting expression levels in terms of Ct values for the seven candidate genes in ten different samples, and across all sample types. Boxes encompass 25th to 75th percentiles. Whisker caps denote maximum and minimum Ct values.

Expression levels were quantified and individual candidate variability in any developmental stage, cell culture, and throughout all assays are summarized as Box-Whisker plots in Fig. 1. The highest recorded Ct value amongst the seven genes was by *RPS17* at 15.02, whereas the lowest was by *α*-*tubulin* at 33.55. *RPS17* and *α*-*tubulin* each produced the highest and lowest mean Ct values of 17.86 and 23.83, respectively, when all sample types (n = 17) were taken into account. The other candidate genes were also expressed at high moderate to low moderate levels, with mean Ct values of 18.94, 19.61, 19.71, 20.12, 21.32, and 23.65 each to *RPL32, RPL8, eEF1α, ACT*, and *GAPDH*. The target gene *CTPsyn* had a mean Ct of 22.50, with values ranging from 18.99 to 26.32. This means that comparatively, *CTPsyn* displayed a narrower Ct variation than six out of the seven candidate genes i.e. *ACT, eEF1α, α*-*tubulin, GAPDH, RPL8* and *RPL32* (Table 1). Sample integrity is inferred from the intrinsic variation (InVar) score as generated by the BestKeeper algorithm. Removal of samples with scores in excess of ±3.0 is recommended^27^. In Var scores for most of the individual developmental stages were low and did not exceed the proposed exclusion value. Triplicate variability was acceptable for all samples. As expected, InVar scores were found to be excessively high when all Ct values were pooled. This strengthens the opinion that no single gene would satisfy all stages, and that each would likely have a unique set of reference genes most suitable for normalization.

### BestKeeper analysis

BestKeeper estimates the standard deviation (SD) value of each candidate gene from raw Ct numbers. An SD > 1 signifies that the variations in expression of a gene within a sample of the same origin are high, and thus indicating its instability. Our data demonstrated that not all candidates were stable across all samples (see Supplementary Table S2). Expression appears to vary most within adult tissues. All barring *RPS17* were unstably expressed in adult male samples. *RPL8, α*-*tubulin, eEF1α* and *RPL32* showed high SDs in non-blood fed female adults. Additionally, *ACT* was shown to be unstable in 48 to 72 hour embryos; *α*-*tubulin* in 6 to 9 hour embryos; *eEF1α* in 48 to 72 hour embryos and fourth instar larvae; *RPL8* throughout the 24 to 72 hour embryonic periods as well as cell samples; *RPL32* in 24 to 48 hour embryos and both first instar and third instar larvae; and *GAPDH* in 48 to 72 hour embryos, all larval stages, and cell culture. The target gene *CTPsyn* gave relatively low SD values, only exceeding 1 in 48 to 72 hour embryos and Aag2 cells. Ranking of genes is based on the value given as *BestKeeper* vs *Pearson* correlation of coefficient. The closer this value is to 1, the greater the reliability of the gene. A third component of the program’s statistical analysis is a *P*-value, where *P* < 0.05 indicates the correlation of a candidate gene to the *BestKeeper index* calculated as the geometric mean of the Ct values. Our data showed that for all instances where the *BestKeeper* vs *Pearson* correlation of coefficient value is above 0.67, the gene would be significantly correlated to the *BestKeeper index*. With genes carrying SD values of above 1 excluded, *RPS17* is most reliable for the 48 to 72 hour embryos, first instar larvae and adult male stages; *α*-*tubulin* for 0 to 3 hour and 24 to 48 h embryos as well as fourth instar larvae; *eEF1α* for both 6 to 9 hour and 18 to 24 hour embryos, along with third instar larvae; *ACT* for the 9 to 12 hour embryonic stage as well as both non-blood fed and blood-fed adult female samples; *RPL8* for 3 to 6 hour and 12 to 18 hour embryos, second instar larvae, and pupae; and *RPL32* for Aag2 cell culture samples. However, for comparison purposes, all genes are included regardless of SD values. Rankings are shown in Table 2.

### geNorm analysis

**Figure 2:**
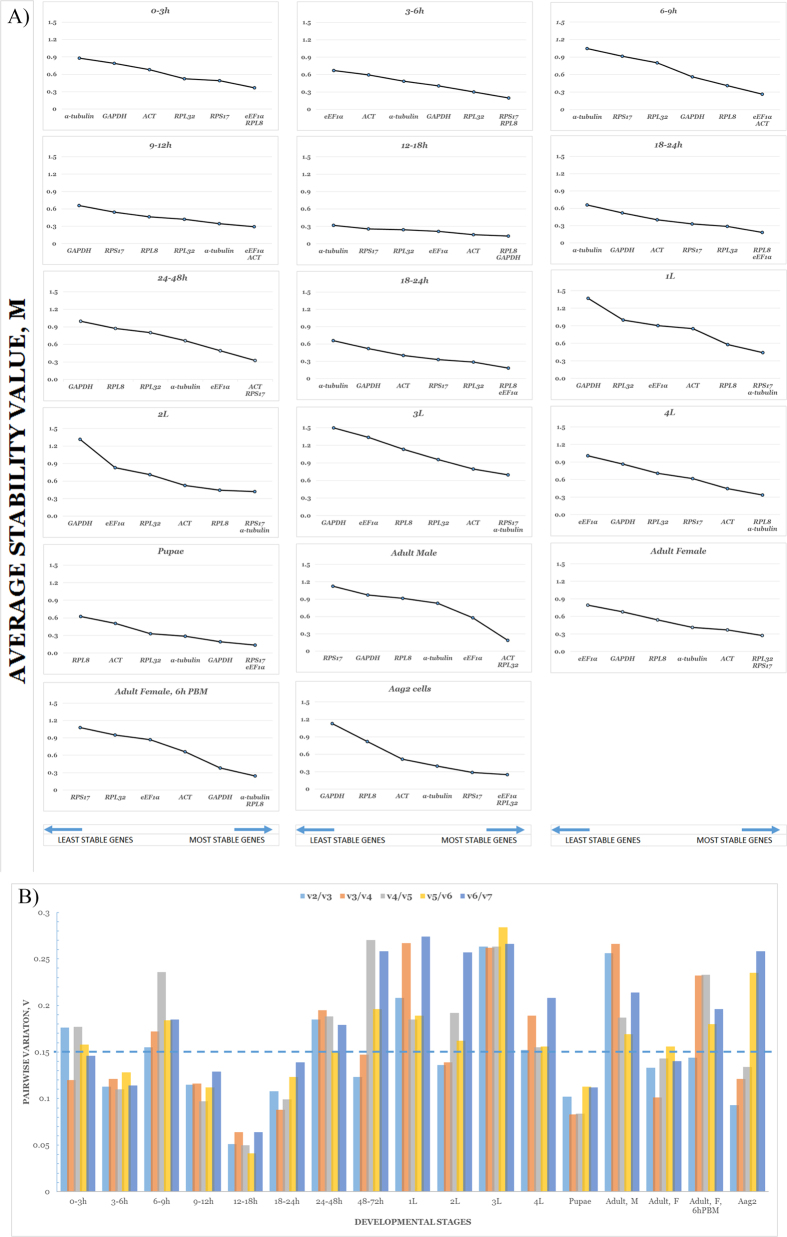
(**A**) Average stability values (M) of genes in individual developmental stages and cell culture. (**B**) Pairwise variation (V) analysis of candidate reference genes. For each Figure the graphs represent (**A**) 0 to 3 hour embryos (**B**) 3 to 6 hour embryos (C) 6 to 9 hour embryos (D) 9 to 12 hour embryos (E) 12 to 18 hour embryos (F) 18 to 24 hour embryos (G) 24 to 48 hour embryos (H) 48 to 72 hour embryos (I) First instar larvae (J) Second instar larvae (K) Third instar larvae (L) Fourth instar larvae (M) Pupae (N) Adult Male (O) Adult Female (P) Adult Female, 6 hours Post-Blood Meal (Q) Aag2 cells.

geNorm determines the expression stability of selected candidate genes based on a data comprised of relative values, i.e. the degree of fold differences observed between Ct values of a sample set in relation to the lowest recorded value. Two assessment outcomes are provided by the software. The first is an average expression stability score as symbolized by M. The higher the M-value of a gene, the less stable it is perceived to be. This value should fall below 1.5. Rankings based on the M-value are in Table 3. Single-normalizer strategies can reliably apply *RPL8* or *eEF1α* for embryo-derived samples of any time point within the first 24 hours, and either *ACT* or *RPS17* for samples from 24 through to 72 hours. *RPS17, α*-*tubulin* or *RPL8* appear suitable for larval stages; *RPS17* or *eEF1α* for pupae samples; *ACT* or *RPL32* for adult stages, and *eEF1α* or *RPL32* for cell culture samples. A summarization of rankings in charts as provided by the software is as shown in Fig. 2A. The second outcome from geNorm is a pairwise variation or V value which estimates the effect of a gene addition event^10^. The proposed cut-off value is 0.15. Our data showed that for ten out of the seventeen sample types, two reference genes may be enough for normalization of target gene expression (Fig. 2B). In 0 to 3 hour embryos, the addition of a third gene is recommended for proper normalization. For the other developmental stages, none of the gene inclusion events resulted in satisfactory V values.

### NormFinder analysis

Similar to geNorm, the data utilized by this program is based on relative values, and not raw Ct data. The algorithm produces a stability value for each gene where a lower value indicates greater stability. The program does not make suggestions for a cut-off value^28^. Rankings formed by NormFinder are summarized in Table 4. *ACT* and *eEF1α* are interchangeable in terms of usability as the reference gene for embryos aged between 0 to 48 hours and all adult samples, whereas *RPL32* is best for embryos aged between 48 and 72 hours. *RPS17* is the best performing gene in both first and second instar larvae as well as pupae; *α*-*tubulin* for the third and fourth larval stages, and *eEF1α* for cell culture samples. *GAPDH* and *α*-*tubulin* are not recommended for embryonic, larval nor cell samples, and *RPS17* should not be the normalizer for adults. In the absence of group identifiers, it is presumed that the two genes with the lowest stability value within a sample set would provide the best combination for two-reference gene normalization strategies^29^.

### Consensus list of reference genes

Consensus rankings are obtained through geometrically averaging the weights assigned to each gene (in the form of stability values from geNorm and NormFinder, and a function of 1-((*BestKeeper* vs. *Pearson* correlation coefficient value) from BestKeeper) as generated by the three programs. All genes are included regardless of BestKeeper SD values. Results are summarized in Table 5. The three top-ranked genes in the consensus list for any developmental stage are typically considered to be most reliable e.g. *eEF1α, RPS17, GAPDH* for pupal samples; *α*-*tubulin, RPL8* and *RPS17* for both the third and fourth instar stages; and *RPL32* alongside*eEF1α* and*RPS17* in Aag2 cell culture samples. However, as these vary greatly from one developmental stage to the next, reliability across sample types is also assessed on the basis of overall frequency at which a gene appears amongst the top-three. *ACT* and *RPS17* are the most reliable, with a frequency of 0.216(11/51) and 0.196 (10/51), respectively.

### Validation of consensus rankings using CTPsyn target gene

To evaluate consensus ranking outcomes, an assumptive analysis was undertaken. Relative expression of the target gene *CTPsyn* was investigated ineach sample type. This gene was chosen due to its expected expression stability. Therefore, it is assumed that the ‘true’ fold change value for any developmental stage is derived solely from the △Ct of *CTPsyn* (2^ΔCt^_CTPsyn_). Fold-change as predicted by a normalizing gene(s) is calculated with the Livak method i.e. 2^−ΔΔCt^ (see Supplementary Table S3). Outcomes of normalization against single, top-two consensus-ranked, top-three consensus-ranked, as well as *ACT/RPS17* gene combinations are as shown in Table 6. For thirteen out of the sixteen developmental stages as well as cell samples, a single-normalizer strategy estimated *CTPsyn* fold-change most effectively. *RPS17* was the best performing gene for six points of development including in blood-fed females, despite being ranked last within its consensus. *RPL8, RPL32* and *α*-*tubulin* are each the best normalizer for two, whereas *eEF1α* and *GAPDH* each normalized best for one developmental stage. This suggests that if chosen carefully, application of one reference gene may be sufficiently robust for gene expression evaluation. Our analysis also demonstrated that the usage of sequentially ranked genes in combinations of three is preferable to two in estimating fold-change values for most sample types. However, an *ACT/RPS17* pairing outperformed both two and three-gene combinations in eleven developmental stages as well as cell culture, suggesting that these two genes together could provide proper normalization regardless of sample origin.

## Discussion

As qPCR increasingly becomes the method of choice in gene expression-focused studies, the need for reliable reference genes grows ever more urgent^6^,^8^. Misinformed selection of reference genes could lead to false positives or false negatives, effectively masking the true nature of a gene’s expression patterns^30^. Though *Ae. aegypti* has been recognized as an important vector of viral diseases for years, it is only recently that research involving the insect has shifted focus to the host back from the viruses it transmits. The *Ae. aegypti* genome is now fully sequenced and annotated^31^, and its developmental transcriptome is described^32^. Nonetheless, studies with *Ae. aegypti* involving qPCR often adopt genes already exhaustingly utilized as the reference genes for insects of other genera for normalization purposes. In most, the lack of dependable information regarding reference genes for *Ae. aegypti* has limited normalization to be against a singular reference gene, a practice which could lead to inaccurate data interpretation^33^,^34^. Now that it is thrusted into the spotlight as a major proponent of global epidemic threats, in-depth molecular research into the mosquito’s life cycle is vigorously ongoing and thus, it is high time that a detailed panel of reference genes uniquely catering *to Ae. aegypti* is defined.

In this study, a total of sixteen candidate genes including *18S, ATP5C1, PGK1, TBP* and *RNAPII* were initially identified as candidates. However, these were gradually eliminated due to several factors, including the lack of introns; primer design difficulties due to consecutive base runs spanning exon-exon boundaries; and low basal expression levels (Ct ≥ 30) even at a high starting cDNA concentration discommending the gene from usage as a reference. Remaining genes such as ribosomal proteins *RPL8, RPS17* and *RPL32* were chosen on the basis of frequency of appearance in *Ae. aegypti* literature^19^,^21^,^35^,^36^,^37^,^38^. *GAPDH, ACT, α*-*tubulin* and *eEF1α* were selected as available transcriptome data have demonstrated their level expression across the diverse developmental states of this insect^32^.

Our study sampled subgroups comprising of sixteen pre-determined stages of development in *Ae. aegypti* as well as Aag2cells. Seven candidate genes were selected and their stability within each developmental stage is evaluated. Our data resulted in three differing rankings from the three evaluation programs employed, i.e. BestKeeper, geNorm, and NormFinder. Nonetheless, some degree of congruence was clearly demonstrated. Results from geNorm and NormFinder were similar in seven developmental stages as well as cell culture. The rankings generated from BestKeeper and NormFinder were alike in 3 to 6 hour and 18 to 24 hour embryos, whereas overlaps were seen between BestKeeper and geNorm in the 12 to 18 hour embryonic samples and blood-fed female adults. Good overall congruency was observed for male and non-blood fed female adults across all three programs. Conversely, major disagreements appeared in candidate gene rankings in 48 to 72 hour embryos (see Supplementary Table S3). As a whole, results generated by BestKeeper tended to contradict those of geNorm and NormFinder both, as was especially noticeable for results in second and third instar larvae. Disparities are to be expected, as each program is based off its own unique algorithm^39^,^40^. Moreover, many of the candidate genes in this study fall within the same functional groups. Algorithmic dissimilarities and the resulting differences in sensitivity each program would have towards co-regulated reference genes may have led to this observation^10^,^27^,^28^,^41^. BestKeeper also has more considerations in the form of InVar, SD, and *P*-values, all of which contribute to the *BestKeeper* vs. *Pearson* correlation coefficient value. These compounding factors result in the obvious differences in final outcomes.

Findings additionally reiterate the notion that there is no universal reference gene stable enough to counterbalance all age and developmental-point imposed variations in gene expression^42^. The candidate genes investigated here showed considerable variation in expression across different samples. Four displayed a range exceeding 10 Ct, with total standard deviation values of above ±2.2. Most exhibited a larger Ct value range than the target gene used for validation i.e. *CTPsyn*. Although this could be due to the possibility that *CTPsyn* – as a synthetase enzyme producing the constantly in-demand CTP molecule – may on its own be a reference gene, the significantly high standard deviations as seen in several samples emphasizes the necessity for an assessment of reference genes in accordance to situational parameters. However, it is impractical to evaluate a large number of candidate genes for every minor qPCR procedure. With developmental stages grouped by tissue and morphological characteristics, the consensus ranking suggests certain two-gene combinations for normalization. Throughout the critical first 24 hours of embryonic development, a pairing between *eEF1α* and either *ACT* or *RPL8* is optimum. A combination of *RPS17* and *ACT* is best for embryos aged 24 to 72 hours. *RPS17* could also provide ideal normalization when simultaneously applied with *α*-*tubulin* during the first, third and fourth instar larval stages; with *RPL8* in second instar larvae; and with *eEF1α* with pupal tissue. *ACT* along with *α*-*tubulin* should normalize adult female expression levels regardless of blood-feeding status, whereas *RPL32* and *eEF1α* are suggested for samples of male adults and Aag2 cells. The genes least recommended as reference for egg or embryo-derived tissue samples are *α*-*tubulin* and *GAPDH*. For any larval stage, the usage of *RPL32, eEF1α* or *GAPDH* is unadvisable. *RPL32* should be avoided when evaluating gene expression in samples from pupal tissue; *RPS17* in male and blood fed female adult tissue; and *GAPDH* in samples derived from non-blood fed female adults as well as Aag2 cells. The frequency with which *GAPDH* appeared at the lower end of consensus rankings was surprisingly high, as the reliability of the gene as a reference in arthropods has been shown time and time again^43^,^44^,^45^. Nevertheless, given the high standard deviation and overall instability of gene as displayed by our data, we are comfortable in suggesting caution when utilizing *GAPDH* in regards to *Ae. aegypti*.

When normalizing expression of a target gene against a reference(s), the objective is to minimalize the normalization factors (NF) value. In geNorm, stepwise inclusion of the next best gene is given as a score denoted as pairwise variation or V value. When this falls below a 0.15 threshold, it suggests that the addition event will only slightly contribute towards decreasing the NF value, and may thus be unnecessary. The V values as generated from our data indicate that in ten out of the seventeen sample subgroups, the combination of two genes should be sufficient for normalization. However, our analysis with the target gene *CTPsyn* demonstrated that a top-two gene combination was the best normalizer only for second instar larval tissue. Although this same strategy performed well in general for all developmental stages and cell culture, in fourteen of the seventeen subgroups, a singular gene adequately provided proper normalization, though the most effective gene in these instances is never the top-ranked gene. This pairwise variation analysis additionally displayed that no consecutive gene inclusion events satisfied the 0.15 threshold value in 6 to 9 hour embryos, 24 to 48 hour embryos, first, third and fourth instar larvae, as well as adult male tissue samples. Several studies have suggested that as the threshold cutoff point serves as a guide rather than a rule, observing the changing trends as gene inclusion proceeds is more indicative of the ideal number of normalizing genes than the actual value themselves^46^,^47^. Others propose that the utilization of the three genes with the lowest M values should most appropriately assist with normalization^10^,^48^,^49^,^50^. It is therefore of great interest to note that when validated against *CTPsyn*, triple-combos of top-ranked genes consistently gave more accurate estimations of fold-change than double-combos, and together with findings from previous studies^41^,^51^, indicate that applying the suggested minimum of three genes may simultaneously be the most practicable and useful strategy.

Although certain candidates may indeed be able to serve as the sole reference gene for certain developmental stages, our validation analysis also showed that normalization power across most samples did not adhere to its consensus ranking. Top genes often deviate quite significantly from the ‘true’ fold-change value on their own. Selecting the most preferable reference gene may thus devolve into a matter of guesswork and luck. Furthermore, as the data set becomes vulnerable to the variables encountered along the qPCR process^10^,^17^, dependence on a singular reference gene for normalization remains an undesired practice. This is especially true when considering the complex nature of a sample set such as ours. The sixteen developmental stages in this study represent a series of transitions in the *Ae. aegypti* growth environment i.e. from terrestrial (egg or embryo), to aquatic (larval and pupal stages), and back to terrestrial (adult). Though in theory a ‘reference’ gene should not be influenced by such circumstantial stress, this is often not the case. Changes undergone by the organism throughout these periods may exacerbate gene expression variability amongst the sample subgroups. This weakens the gene’s stability, thus impairing its ability to reduce the NF value. Such an observation supports the claim that the suitability of reference genes could be experimentally exclusive, and that a panel of candidates should be simultaneously assessed within the confines of the variables of an assay.

As aforementioned, this may not however be feasible in scientific practice. Therefore, we proposed *ACT/RPS17* combination for general usage in normalization practices for *Ae. aegypti*, regardless of tissue sample origins. This pair of genes appeared with the highest frequencies within the consensus top-three ranks of this study, alluding to their overall stability and dependability in countering developmentally-influenced variation. *ACT* encodes for a component of the cytoskeleton. Its importance in upholding structural integrity ensures that the gene is expressed at moderately high levels within every cell type. Over the years, *ACT* has acquired a rather bad reputation due to several instances whereby its seemingly excessive usage as a reference gene was proven to be unjustified. Regardless, *ACT* has been ranked as the most stable reference gene in a number of validation studies^43^,^52^,^53^. The gene *RPS17* produces S17, a protein component of the 40S ribosomal subunit. It has long been used as the reference gene in *Ae. aegypti* transcriptional profiling^36^,^54^,^55^,^56^, and is relied upon as the reference gene in numerous studies involving insects^57^,^58^,^59^. Readily available transcriptomics data exhibited minimal *ACT* expression variation throughout *Ae. aegypti* development^32^. However, their levels within the mosquito head appear to be moderately affected by rhythmic circadian cycle changes^60^. Conversely, the same studies reported rather significant *RPS17* fluctuations during developmental progression, but minimal changes due to light-dark switches. These clashes in situations where *ACT/RPS17* are more likely to vacillate perhaps allows the genes to counterbalance one another as they act together to normalize gene expression.

Nonetheless, improvements could be made in the future through increasing the number and characteristic variability of candidate genes in further reference gene validation studies. In *Drosophila*, it has been shown that a larger panel of reference genes is required as the sample size and inherent complexity grows^61^,^62^,^63^. Selection of gene types is also crucial. Here, the candidate genes can be separated into only three categories: (i) genes linked to ribosomal functions and/or protein production, (ii) structural genes producing components of the cellular protein scaffold, and (iii) metabolism-related genes. In the future, including other common reference genes for evaluation such as genes encoding ubiquitin proteins, phosphatases e.g. *PP2A*, and oxygen-radical metabolizing proteins e.g. *SOD* and *CAT*, could improve normalization and consequently the integrity of gene expression studies in *Ae. aegypti*^64^,^65^,^66^.

## Conclusion

To our best knowledge, this is the first study of its kind in *Ae. aegypti*. Through the utilization of algorithms specifically conceptualized for reference gene validation, a suitable panel of genes most robust for normalization are identified for each developmental stage and Aag2 cell culture. Our results show that although a singular reference gene may suffice for interpretation of target gene expression in most stages, a combination of at least two genes is recommended to minimize the effects of variables upon the data set and for consistency of normalization. Application of three genes for normalization is optimum. Based on consensus rankings, the proposed combinations are *RPL8, ACT* and *eEF1α* for early embryos between 0 to 24 hours post-oviposition; *ACT, RPS17* and *eEF1α* for embryos 24 to 48 hours of age; *RPS17, RPL32* and *ACT* for late embryos (aged between 48 to 72 hours); *RPS17, α*-*tubulin*, and *ACT* are recommended for first instar larvae; *RPS17, RPL8* and *GAPDH* for second instar larvae; *α*-*tubulin, RPL8* and *RPS17* for both third and fourth instar larval samples; *eEF1α, RPS17* and *GAPDH* for pupal samples; *RPL32, eEF1α* and *ACT* for adult male tissue; *ACT, α*-*tubulin*, and *RPS17* for non-blood fed female adults; *ACT, α*-*tubulin* and *RPS17* for blood-fed female adults; and finally, *RPL32* along with *eEF1α* and *RPS17* for Aag2 cell samples. *GAPDH* is ranked lowest for most developmental stages, and is thus not to be used as a reference gene. Overall, inferred stability suggests *ACT* and *RPS17* as the most dependably expressed reference genes and therefore, an *ACT/RPS17* combination is expected to provide robust normalization for genetic expression studies in all *Ae. aegypti* derived samples. These findings will benefit normalization practices in *Ae. aegypti*, and may additionally serve as a resource for screening reference genes in closely-related arthropods.

## Materials and Methods

### Rearing and sample collection

About 500 dried viable eggs of VCRU-lab strain *Ae. aegypti* were obtained from Vector Control Research Unit, Universiti Sains Malaysia and hatched in dechlorinated water. Larvae were maintained in relative humidity and natural light conditions at 28 °C in plastic containers. Rearing water is changed every other day. They were fed daily with crushed baby biscuits (Milna™ Rusks, Kalbe^®^). Larval samples collected were of first (1 L; 80 individuals), second (2 L; 60 individuals), third (3 L; 40 individuals), and fourth (4 L; 20 individuals) instar stages. Pupal samples were a mixture of an equal number of individuals at first, second, and third day of pupation (5 of each day; total of 15 per bioreplicate). Newly-eclosed adults were maintained in cages and fed on 10% sucrose solution. Adult samples collected comprised of equal numbers of males and non-blood fed females aged 1 to 10 days after eclosion (DAE) (two of each gender at each DAE; 20 total per bioreplicate). Food was removed from 5 to 7 DAE adults a full day before being blood-fed using artificial membrane blood feeding system. 20 females were collected 6 hours after the blood meal as post-blood meal samples. The remaining mosquitoes were returned to normal conditions for females to lay eggs. Around 250 embryos were collected at each of the following time points post-oviposition: 0 to 3 hours (0–3 h), 3 to 6 hours (3–6 h), 6 to 9 hours (6–9 h), 9 to 12 hours (9–12 h), 12 to 18 hours (12–18 h), 18 to 24 hours (18–24 h), 24 to 48 hours (24–48 h), and 48 to 72 hours (48–72 h). Aag2 cells cultured in Gibco^®^ L-15 media supplemented with 10% FBS, 1% Pen-Strep and 10% Tryptose Phosphate Broth (all manufactured by ThermoFisher Scientific, USA) in a non-CO_2_ incubator at 28 °C were harvested at maximum confluency. Three biological replicates were collected for each developmental stage as well as cell culture.

### RNA extraction and quality assurance

This study attempts to adhere to the Minimum Information for Publication of Quantitative Real-Time PCR guidelines or MIQE^17^. Samples collected throughout rearing were immediately stored in TRIzol^®^ reagent (Invitrogen™, Ambion™, Life Technologies) at −20 °C. Total RNA extraction was done within two days of collection with a protocol previously described for mosquito tissue samples^67^. As much of the culturing media was aspirated away from Aag2 samples, prior to RNA extraction as described by Abcam^®^^68^. Extracts were quantified on the Hellma^®^ Analytics TrayCell system in the SmartSpec Plus Spectrophotometer (Bio-Rad Laboratories, California). Those with an A260:A280 value between 1.75 and 2.05 were used immediately for downstream procedures. All showed clear 18S banding and minimal smearing in 1.0% agarose gel. An RNA gel with randomly chosen first bioreplicate extracts of differing degrees of freshness is shown as Supplementary Figure S1A. Extracts were kept at −20 °C for the duration of the experiment.

### Reference gene selection, primer design, and primer validation

All genes are also commonly utilized reference genes in qPCR protocols. Primers for *RPS17* were previously described^19^. Others were designed on the Primer 3 software (bioinfo.ut.ee/primer3–0.4.0/). Restrictive parameters for primer selection were: melting temperatures between 59.0 °C and 61.0 °C, GC content between 40 and 60%, nucleotide length between 18 and 24, and amplicon length of between 150 to 225 bases. Regions spanning exon-exon boundaries were specified for each primer pair. Other settings were kept at default. PCR product was confirmed *in silico* on the Sequence Manipulation Suite website (http://www.bioinformatics.org/sms2/) to be a singular amplicon from only the mature mRNA, and not the genomic DNA sequence. All genes, accession numbers, primer sequences and amplicon size used for this study is listed in Table 1.

### Reverse transcription and qPCR

Reverse transcription with 1 μg of total RNA was carried out in 20 μl reactions using the iScript Reverse Transcription Supermix (Bio-Rad Laboratories, California; cat. no. 1708840) according to manufacturer’s protocol. Undiluted cDNA from all nine-developmental stages were pooled and serially diluted to the factor of 5 (1:1, 1:5, 1:25, 1: 125, 1:625 and 1:1875) for standard curve generation on the BioRad CFX96 qPCR platform. Optimum qPCR reactions were carried out in 10 μl reactions using iTaq™ Universal SYBR^®^ Green Supermix (Bio-Rad Laboratories, California; cat. no. 1725120), ~1 ng total cDNA, and 500 nM each of forward and reverse primers. The standard run protocol is initial denaturation at 95 °C for 2.30 mins, followed by 40 cycles of denaturation at 95 °C for 20 s, annealing at 59 °C for 20 s and extension at 72 °C for 15 s. After a final extension at 72 °C for 20 s, the machine would perform a melting-curve analysis. All samples were amplified in technical triplicates. Expression levels were recorded as cycle quantification (Cq). Efficiency values (E) were calculated according to the equation: E = (10^[−1/slope]^−1) × 100^69^.

### Data mining and selection of reference gene candidates with algorithms: geNorm, BestKeeper, and NormFinder

To assess the stability of candidate reference genes, publicly available evaluation tools i.e. BestKeeper (http://www.gene-quantification.com/bestkeeper.html)^27^,^70^, geNorm^10^ and Normfinder^28^ were utilized. The BestKeeper algorithm has been adapted for usage in Excel. The program generates a ranking through repeated pairwise correlation and regression analysis of a gene against all the other tested candidates. Up to ten genes can be evaluated at any one time for expression variations in up to one hundred samples. Raw data of Ct values (annotated as CP) and PCR efficiency of the primers were used to determine the correlation between each candidate gene and the index, expressed in the form of a coefficient of determination^70^. For geNorm and NormFinder, raw data was converted into linear values relative to the lowest Ct recorded for each candidate gene. In geNorm, the stability of a gene is assessed through the consistency of its expression ratio across all samples. The software generates both a stability value i.e. M, and a pairwise variation value i.e. V. M represents the average variation in transcript levels of a gene in comparison to all other candidate genes, achieved through a repeated process of stepwise exclusion commencing from the least stable gene. Pairwise variation estimates the effect of including another gene^10^ sequentially as per the established M-value rankings through the formula of V_n_/V_n_ + 1. A threshold of 0.15 is set; a V value below this would mean that an additional reference gene would not improve normalization. NormFinder is a mixed-effects model statistical analysis which estimates the stability value of a gene as a function of the approximate expression variation it would impose onto the target gene data during normalization^28^. The lower this value is, the less variation one would introduce to a normalization exercise should the candidate gene be used as a reference. It also estimates the variation between sample subgroups of the sample set. The *BestKeeper* vs. *Pearson* correlation coefficient value, geNorm M value, and NormFinder stability value are perceived as weightage. Geometric means i.e. central tendencies of these weightages for a candidate gene forms the basis for generation of a consensus ranking.

### Evaluation of results through target gene normalization

*CTPsyn* of *Ae. aegypti* was utilized as the target gene for candidate reference gene evaluation. This gene encodes for the enzyme CTP Synthetase, which converts UTP, ATP and glutamine into cytidine triphosphate (CTP) molecules^71^. Human isoforms of *CTPsyn* have been identified as potential reference genes^72^. Although the expression levels of the gene in insects and *Ae. aegypti* in particular are undefined, as a housekeeping gene, it is expected to be expressed stably across all developmental stages. For this reason, *CTPsyn* is chosen as the ‘target’ gene for the purpose of this analysis. Primer sequences for *CTPsyn* were forward 5′TTCCCCATTGCTACCCGAAC and reverse 5′GAAAACCCTTCCCCAGCGTA. The expected product size is 180 bp. ‘True’ fold-change is based solely on *CTPsyn*, in the function of 2^−ΔCt^_CTPsyn_. Fold changes estimated from normalization with (a) different genes, and (b) differing combinations and number of genes was calculated according to the Livak method i.e. 2^−ΔΔCt^ 73. The degree of difference between the value predicted by normalizer(s) against fold change of *CTPsyn* △Ct is the basis for evaluation of the effectiveness of the candidate reference gene(s) in normalization.

## Additional Information

**How to cite this article:** Dzaki, N. *et al*. Evaluation of reference genes at different developmental stages for quantitative real-time PCR in *Aedes aegypti. Sci. Rep.* **7**, 43618; doi: 10.1038/srep43618 (2017).

**Publisher's note:** Springer Nature remains neutral with regard to jurisdictional claims in published maps and institutional affiliations.

## Supplementary Material

Supplementary Information

## Figures and Tables

**Table 1:** Specifications and amplification characteristics of candidate genes.

Gene	AAEL# ID (GenBank BankIt No.)	Primer sequence	Amplicon size (bp)	Ct range	Std. Error	R2	E%
*ACT*(*Actin1*)	AAEL011197 (KY000701)	FW 5′ CGTTCGTGACATCAAGGAAA	175	17.78–27.70	1.774	0.996	97.2
		RV 5′ GAACGATGGCTGGAAGAGAG					
*a*-*tubulin*(*Alpha*-*Tubulin*)	AAEL013229 (KY000707)	FW 5′ CTGCTTCAAAATGCGTGAAT	225	19.61–33.55	2.248	0.979	100.8
		RV 5′ GGTTCCAGATCGACGAAA					
*RPS17*(Ribosomal Protein S17)[19]	AAEL004175 (KY000705)	FW 5′ AAGAAGTGGCCATCATTCCA	200	15.02–21.02	1.126	0.997	96.7
		RV 5′ GGTCTCCGGGTCGACTTC					
*RPL8*(*Ribosomal Protein L8*)	AAEL000987 (KY000704)	FW 5′ AAGGGAGAGCCAAAATTGC	200	15.29–28.15	2.298	0.981	96.8
		RV 5′ CAGTACACAAACTGTCCGGTGT					
*RPL32*(*60S Ribosomal Protein L32*)	AAEL003396 (KY000706)	FW 5′ CAGTCCGATCGCTATGACAA	200	16.08–26.08	1.720	0.995	100.8
		RV 5′ ATCATCAGCACCTCCAGCTC					
*GAPDH*(*Glyceraldehyde 3*-*phosphate dehydrogenase*)	AAEL016984 (KY000703)	FW 5′ ACAGACGCTAGTTATCAACGTA	194	18.44–32.20	2.797	0.989	92.5
		RV 5′ ACCGTGGGTCGAATCGTA					
*eEF1a*(*Eukaryotic Elongation Factor1Alpha*)	AAEL017301 (KY000702)	FW 5′ AGGAATTGCGTCGTGGATAC	218	15.98–27.00	2.210	0.996	95.3
		RV 5′ GTTCTCTTCGGTCGACTTGC					

**Table 2:**
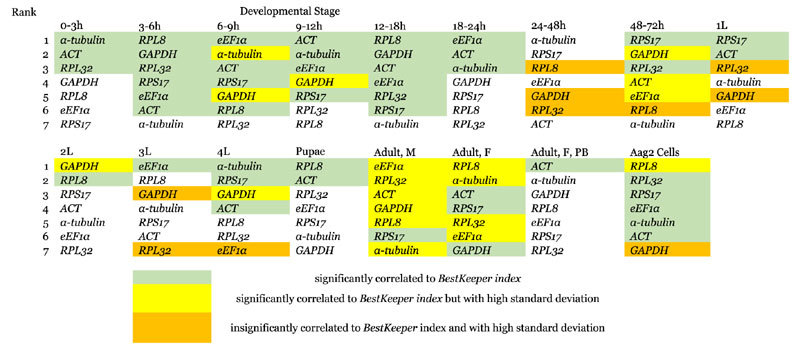
Rankings of candidate genes by BestKeeper.

Rankings are determined by *BestKeeper* vs *Pearson* correlation coefficient (R) values. The closer the value is to 1, the greater the reliability of the gene.

**Table 3:** Rankings of candidate genes by geNorm.

Rank	Developmental stage								
	0–3 h	3–6 h	6–9 h	9–12 h	12–18 h	18–24 h	24–48 h	48–72 h	1 L
1/2	eEF1α/RPL8	RPL8/RPS17	ACT/eEF1α	ACT/eEF1α	RPL8/GAPDH	eEF1α/RPL8	ACT/RPS17	ACT/RPL8	RPS17/α-tubulin
3	RPS17	RPL32	RPL8	α-tubulin	ACT	RPL32	eEF1α	RPS17	RPL8
4	RPL32	GAPDH	GAPDH	RPL32	eEF1α	RPS17	α-tubulin	RPL32	ACT
5	ACT	α-tubulin	RPL32	RPL8	RPL32	ACT	RPL32	α-tubulin	eEF1α
6	GAPDH	ACT	RPS17	RPS17	RPS17	GAPDH	RPL8	eEF1α	RPL32
7	α-tubulin	eEF1α	α-tubulin	GAPDH	α-tubulin	α-tubulin	GAPDH	GAPDH	GAPDH
**Rank**	**Developmental stage**								
	**2 L**	**3 L**	**4 L**	**Pupae**	**Adult**, **M**	**Adult**, **F**	**Adult**, **F**, **6hPBM**	**Aag2 cells**	
1/2	RPS17/α-tubulin	RPS17/α-tubulin	RPL8/α-tubulin	RPS17/eEF1α	ACT/RPL32	RPL32/RPS17	α-tubulin/RPL8	eEF1α/RPL32	
3	RPL8	ACT	ACT	GAPDH	eEF1α	ACT	GAPDH	RPS17	
4	ACT	RPL32	RPS17	α-tubulin	α-tubulin	α-tubulin	ACT	α-tubulin	
5	RPL32	RPL8	RPL32	RPL32	RPL8	RPL8	eEF1α	ACT	
6	eEF1α	eEF1α	GAPDH	ACT	GAPDH	GAPDH	RPL32	RPL8	
7	GAPDH	GAPDH	eEF1α	RPL8	RPS17	eEF1α	RPS17	GAPDH	

Rankings are determined by M values, which should not exceed 1.5. An M score of above this cut-off point suggests overall instability and thus, unsuitability of the gene for usage as a reference gene for the experimental setting. The two top genes share the same value.

**Table 4:** Rankings of candidate genes by NormFinder.

Rank	Developmental stages								
	0–3 h	3–6 h	6–9 h	9–12 h	12–18 h	18–24 h	24–48 h	48–72 h	1 L
1	RPL8	GAPDH	ACT	ACT	eEF1α	eEF1α	ACT	RPL32	RPS17
2	eEF1α	RPL8	eEF1α	RPL32	ACT	ACT	RPS17	RPS17	α-tubulin
3	ACT	RPL32	RPL8	eEF1α	RPL32	RPL8	eEF1α	α-tubulin	RPL8
4	RPS17	RPS17	GAPDH	α-tubulin	RPS17	RPL32	α-tubulin	RPL8	ACT
5	GAPDH	ACT	RPL32	RPL8	GAPDH	GAPDH	RPL8	ACT	eEF1α
6	RPL32	α-tubulin	RPS17	RPS17	RPL8	RPS17	RPL32	eEF1α	RPL32
7	α-tubulin	eEF1α	α-tubulin	GAPDH	α-tubulin	α-tubulin	GAPDH	GAPDH	GAPDH
**Rank**	**Developmental stages**								
	**2 L**	**3 L**	**4 L**	**Pupae**	**Adult**, **M**	**Adult**, **F**	**Adult**, **F**, **6hPBM**	**Aag2 Cells**	
1	RPS17	α-tubulin	α-tubulin	RPS17	eEF1α	ACT	ACT	eEF1α	
2	ACT	RPL8	RPL8	eEF1α	ACT	α-tubulin	eEF1α	RPL32	
3	α-tubulin	RPS17	RPS17	GAPDH	RPL32	RPL32	GAPDH	RPS17	
4	RPL32	eEF1α	ACT	α-tubulin	α-tubulin	RPS17	RPL32	α-tubulin	
5	eEF1α	ACT	RPL32	ACT	RPL8	RPL8	α-tubulin	ACT	
6	RPL8	RPL32	GAPDH	RPL32	GAPDH	eEF1α	RPL8	RPL8	
7	GAPDH	GAPDH	eEF1α	RPL8	RPS17	GAPDH	RPS17	GAPDH	

Rankings are based on scores depicting stability values; the lower the value, the greater the stability.

**Table 5:** Rankings from all three algorithms and resulting consensus.

Rank	Developmental stage								
	0–3 h	3–6 h	6–9 h	9–12 h	12–18 h	18–24 h	24–48 h	48–72 h	1 L
1	RPL8	RPL8	eEF1α	ACT	RPL8	eEF1α	ACT	RPS17	RPS17
2	eEF1α	GAPDH	ACT	eEF1α	GAPDH	ACT	RPS17	RPL32	α-tubulin
3	ACT	RPS17	RPL8	α-tubulin	ACT	RPL8	eEF1α	ACT	ACT
4	RPL32	RPL32	GAPDH	RPL32	eEF1α	GAPDH	α-tubulin	RPL8	RPL8
5	GAPDH	ACT	RPS17	RPL8	RPL32	RPL32	RPL8	GAPDH	RPL32
6	RPS17	eEF1α	RPL32	RPS17	RPS17	α-tubulin	RPL32	α-tubulin	eEF1α
7	α-tubulin	α-tubulin	α-tubulin	GAPDH	α-tubulin	RPS17	GAPDH	eEF1α	GAPDH
	2 L	3 L	4 L	Pupae	Adult, M	Adult, F	Adult, F, 6hPBM	Aag2	
1	RPS17	α-tubulin	α-tubulin	eEF1α	RPL32	ACT	ACT	RPL32	
2	RPL8	RPL8	RPL8	RPS17	eEF1α	α-tubulin	α-tubulin	eEF1α	
3	GAPDH	RPS17	RPS17	GAPDH	ACT	RPS17	GAPDH	RPS17	
4	ACT	eEF1α	ACT	ACT	RPL8	RPL32	RPL8	RPL8	
5	α-tubulin	ACT	GAPDH	α-tubulin	GAPDH	RPL8	eEF1α	α-tubulin	
6	eEF1α	GAPDH	RPL32	RPL32	α-tubulin	eEF1α	RPL32	ACT	
7	RPL32	RPL32	eEF1α	RPL8	RPS17	GAPDH	RPS17	GAPDH	
		Frequency of appearance in top three							
		ACT	eEF1α	α-tubulin	RPL8	RPL32	RPS17	GAPDH	
		0.216	0.157	0.118	0.157	0.059	0.196	0.098	

Consensus rankings are based on the geometric means of weightages in the form of stability values from geNorm and NormFinder, and a function of 1-(*BestKeeper* vs. *Pearson* correlation coefficient value) from Best Keeper.

**Table 6:**
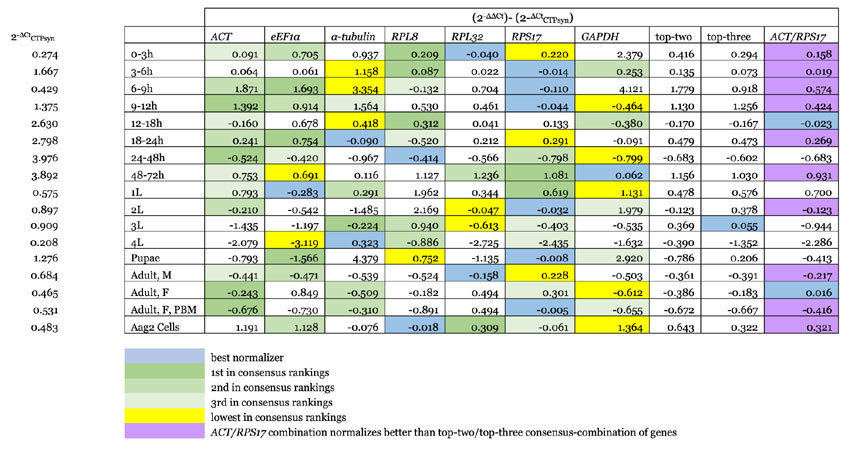
Degree of difference between ‘true’ CTPsyn fold-change value avs as estimated with either single-normalizer genes, or different combinations of top-ranked genes, as denoted.

2^−ΔCt^_CTPsyn_ is the presumptive ‘true’ fold change. ΔΔCt is the product of change when overall geometric mean (GM) of CTPsyn Ct values is compared to Ct value GM of CTPsyn in individual developmental stages.
