# Supplementary material for: Evaluation of reference genes at different developmental stages for quantitative real-time PCR in Aedes aegypti
Source: Sci Rep. 2017 Mar 16;7:43618. doi: 10.1038/srep43618 (PMC5353741; doi:10.1038/srep43618)
Supplement: Supplementary Information [file srep43618-s1.pdf]

Supplementary information

Evaluation of reference genes at different developmental stages for quantitative real-time PCR in *Aedes aegypti*

Najat Dzaki[1], Karima N. Ramli[1], Azali Azlan[1], Intan H. Ishak[1,2] and Ghows Azzam[1,2]*

[1]*School of Biological Sciences, Universiti Sains Malaysia, 11800 Penang, Malaysia*

2Vector Control and Research Unit, School of Biological Sciences, Universiti Sains Malaysia, 11800 Penang, Malaysia

Supplementary Figure S1A: RNA extracts in 1% agarose

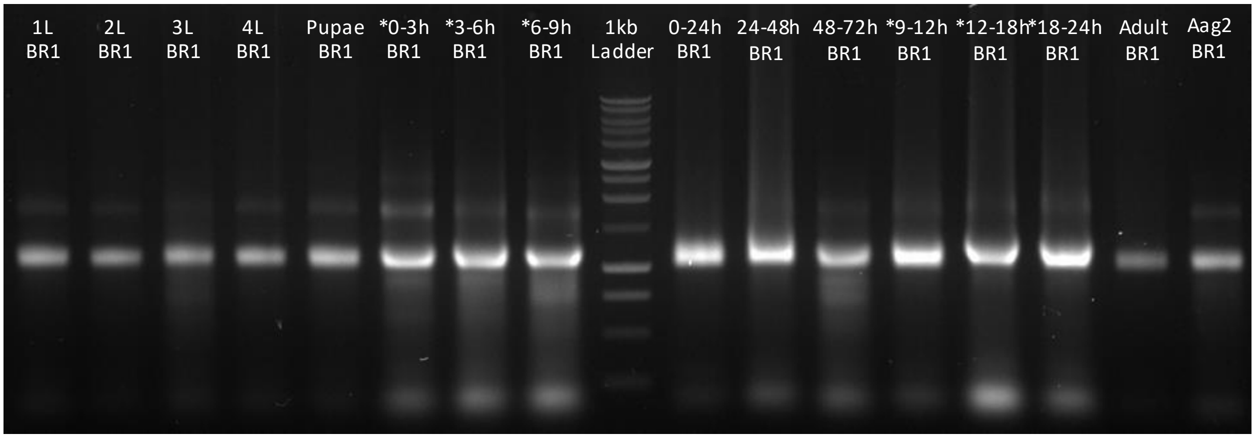

***fresh extracts.** These extracts are less than 4 hours old, and were never frozen. Others not marked with an asterisk are 4-month old extracts, subjected to one to two thaw-freeze cycles, then kept at -20°C. This image is to show that collection of samples in Trizol® does not affect RNA extracts in a negative manner, and that the integrity of larger RNAs is maintained even after long periods of storage; however, the usage of old extracts for small RNA work is not recommended. BR1 = 1st bioreplicate.

Supplementary Figure S1B: qPCR products in 2% agarose

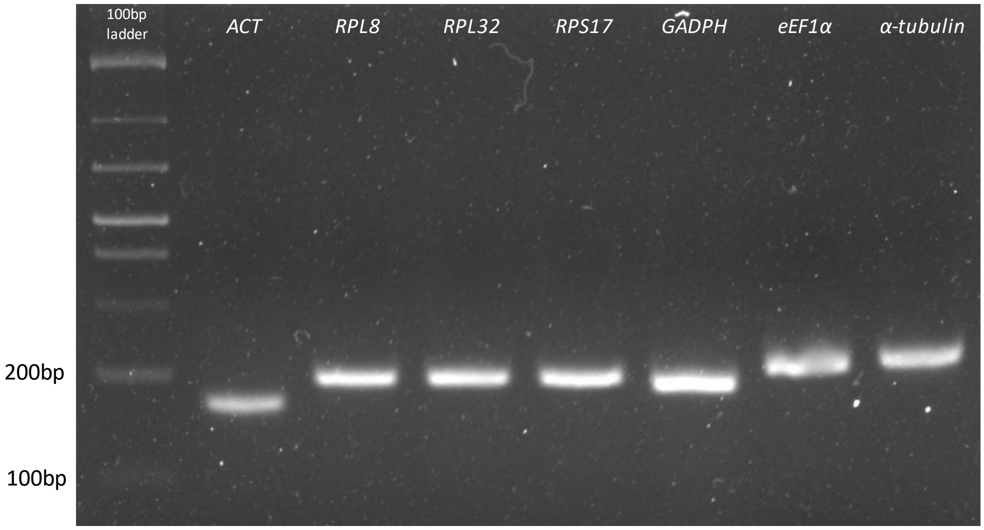

Supplementary Table S1: Sample integrity analysis by BestKeeper. The tables below are as generated and displayed by the program. An InVar score of ±3.0 signifies a three-fold expression level change of the tested candidate gene within the sample, when compared with the overall mean from all samples. To minimalize the effects of a low integrity sample(s), it is recommended to be excluded from further analysis. None of the samples used within this study was variable enough to require exclusion.

(a) 0 to 3 hour embryos

Result - Sample integrity: Variation [InVar] based on selected Housekeeping Genes

print result pages

	n	BestKeeper	InVar. [± CP]	InVar. [± % CP]	InVar. [± x-fold]	InVar. [± CP]	InVar. [± % CP]	InVar. [± x-fold]
sample 1	7	18.93	±0.7	±3.58	±0.41	0.70	3.58	0.41
sample 2	7	18.99	±0.76	±3.89	±0.51	0.76	3.89	0.51
sample 3	7	18.71	±0.95	±4.74	±0.56	0.95	4.74	0.56
sample 4	7	19.19	±0.52	±2.72	±0.45	0.52	2.72	0.45
sample 5	7	19.04	±0.5	±2.67	±0.36	0.50	2.67	0.36
sample 6	7	18.99	±0.49	±2.67	±0.31	0.49	2.67	0.31
sample 7	7	19.41	±0.55	±2.64	±0.58	0.55	2.64	0.58
sample 8	7	19.51	±0.59	±2.76	±0.7	0.59	2.76	0.70
sample 9	7	19.56	±0.62	±2.99	±0.67	0.62	2.99	0.67

(b) 3 to 6 hour embryos

Result - Sample integrity: Variation [InVar] based on selected Housekeeping Genes

print result pages

	n	BestKeeper	InVar. [± CP]	InVar. [± % CP]	InVar. [± x-fold]	InVar. [± CP]	InVar. [± % CP]	InVar. [± x-fold]
sample 1	7	19.43	±0.6	±3.15	±0.39	0.60	3.15	0.39
sample 2	7	19.43	±0.61	±3.13	±0.41	0.61	3.13	0.41
sample 3	7	19.33	±0.41	±2.11	±0.26	0.41	2.11	0.26
sample 4	7	19.07	±0.4	±2.1	±0.25	0.40	2.10	0.25
sample 5	7	18.82	±0.35	±2	±0.16	0.35	2.00	0.16
sample 6	7	18.87	±0.39	±2.14	±0.19	0.39	2.14	0.19
sample 7	7	19.86	±0.49	±2.56	±0.49	0.49	2.56	0.49
sample 8	7	19.82	±0.49	±2.47	±0.5	0.49	2.47	0.50
sample 9	7	19.99	±0.59	±2.94	±0.7	0.59	2.94	0.70

(c) 6 to 9 hour embryos

Result - Sample integrity: Variation [InVar] based on selected Housekeeping Genes

print result pages

	n	BestKeeper	InVar. [± CP]	InVar. [± % CP]	InVar. [± x-fold]	InVar. [± CP]	InVar. [± % CP]	InVar. [± x-fold]
sample 1	7	19.13	±0.82	±4.08	±0.46	0.82	4.08	0.46
sample 2	7	18.98	±0.78	±3.73	±0.38	0.78	3.73	0.38
sample 3	7	18.75	±0.96	±4.62	±0.44	0.96	4.62	0.44
sample 4	7	20.03	±1	±5	±1.03	1.00	5.00	1.03
sample 5	7	20.09	±1	±5.04	±1.06	1.00	5.04	1.06
sample 6	7	19.97	±0.91	±4.51	±0.91	0.91	4.51	0.91
sample 7	7	19.33	±0.56	±2.69	±0.35	0.56	2.69	0.35
sample 8	7	19.60	±0.19	±0.9	±0.14	0.19	0.90	0.14
sample 9	7	19.55	±0.5	±2.46	±0.36	0.50	2.46	0.36

(d) 9 to 12 hour embryos

Result - Sample integrity: Variation [InVar] based on selected Housekeeping Genes

print result pages

	n	BestKeeper	InVar. [± CP]	InVar. [± % CP]	InVar. [± x-fold]	InVar. [± CP]	InVar. [± % CP]	InVar. [± x-fold]
sample 1	7	20.25	±0.62	±3.16	±0.58	0.62	3.16	0.58
sample 2	7	20.03	±0.41	±1.87	±0.36	0.41	1.87	0.36
sample 3	7	20.07	±0.37	±1.72	±0.32	0.37	1.72	0.32
sample 4	7	19.92	±0.32	±1.4	±0.25	0.32	1.40	0.25
sample 5	7	19.74	±0.41	±1.8	±0.33	0.41	1.80	0.33
sample 6	7	19.64	±0.3	±1.34	±0.22	0.30	1.34	0.22
sample 7	7	19.34	±0.65	±3.01	±0.35	0.65	3.01	0.35
sample 8	7	19.36	±0.65	±2.93	±0.35	0.65	2.93	0.35
sample 9	7	19.38	±0.59	±2.63	±0.29	0.59	2.63	0.29

(e) 12 to 18 hour embryos

Result - Sample integrity: Variation [InVar] based on selected Housekeeping Genes

print result pages

	n	BestKeeper	InVar. [± CP]	InVar. [± % CP]	InVar. [± x-fold]	InVar. [± CP]	InVar. [± % CP]	InVar. [± x-fold]
sample 1	7	20.66	±0.36	±1.68	±0.31	0.36	1.68	0.31
sample 2	7	20.65	±0.2	±1.1	±0.17	0.20	1.10	0.17
sample 3	7	20.47	±0.24	±1.14	±0.18	0.24	1.14	0.18
sample 4	7	20.28	±0.13	±0.59	±0.09	0.13	0.59	0.09
sample 5	7	20.19	±0.09	±0.47	±0.06	0.09	0.47	0.06
sample 6	7	20.09	±0.21	±1.11	±0.13	0.21	1.11	0.13
sample 7	7	20.02	±0.27	±1.28	±0.17	0.27	1.28	0.17
sample 8	7	20.04	±0.25	±1.3	±0.16	0.25	1.30	0.16
sample 9	7	20.03	±0.22	±1.03	±0.13	0.22	1.03	0.13

(f) 18 to 24 hour embryos

Result - Sample integrity: Variation [InVar] based on selected Housekeeping Genes

print result pages

	n	BestKeeper	InVar. [± CP]	InVar. [± % CP]	InVar. [± x-fold]	InVar. [± CP]	InVar. [± % CP]	InVar. [± x-fold]
sample 1	7	19.73	±0.65	±2.96	±0.36	0.65	2.96	0.36
sample 2	7	19.73	±0.54	±2.41	±0.29	0.54	2.41	0.29
sample 3	7	19.50	±0.52	±2.3	±0.27	0.52	2.30	0.27
sample 4	7	20.15	±0.14	±0.78	±0.11	0.14	0.78	0.11
sample 5	7	20.00	±0.24	±1.15	±0.18	0.24	1.15	0.18
sample 6	7	19.97	±0.22	±1.02	±0.15	0.22	1.02	0.15
sample 7	7	19.93	±0.56	±2.61	±0.45	0.56	2.61	0.45
sample 8	7	19.98	±0.6	±2.79	±0.51	0.60	2.79	0.51
sample 9	7	19.96	±0.7	±3.41	±0.55	0.70	3.41	0.55

(g) 24 to 48 hour embryos

Result - Sample integrity: Variation [InVar] based on selected Housekeeping Genes

print result pages

	n	BestKeeper	InVar. [± CP]	InVar. [± % CP]	InVar. [± x-fold]	InVar. [± CP]	InVar. [± % CP]	InVar. [± x-fold]
sample 1	7	20.89	±1.11	±4.92	±0.78	1.11	4.92	0.78
sample 2	7	21.07	±0.84	±3.79	±0.63	0.84	3.79	0.63
sample 3	7	21.20	±1.04	±4.85	±1.05	1.04	4.85	1.05
sample 4	7	21.15	±1.69	±7.37	±3.73	1.69	7.37	3.73
sample 5	7	21.30	±1.51	±6.84	±2.35	1.51	6.84	2.35
sample 6	7	21.09	±1.34	±6.33	±1.24	1.34	6.33	1.24
sample 7	7	20.91	±1.1	±5.21	±0.79	1.10	5.21	0.79
sample 8	7	21.21	±1.08	±4.97	±1.16	1.08	4.97	1.16
sample 9	7	21.27	±1.26	±5.7	±1.43	1.26	5.70	1.43

(h) 48 to 72 hour embryos

Result - Sample integrity: Variation [InVar] based on selected Housekeeping Genes

print result pages

	n	BestKeeper	InVar. [± CP]	InVar. [± % CP]	InVar. [± x-fold]	InVar. [± CP]	InVar. [± % CP]	InVar. [± x-fold]
sample 1	7	18.67	±1.1	±5.34	±0.97	1.10	5.34	0.97
sample 2	7	18.85	±1.15	±5.91	±0.76	1.15	5.91	0.76
sample 3	7	18.70	±1.27	±6.72	±0.69	1.27	6.72	0.69
sample 4	7	19.72	±1.39	±6.92	±1.37	1.39	6.92	1.37
sample 5	7	19.67	±1.24	±5.98	±0.97	1.24	5.98	0.97
sample 6	7	19.76	±1.19	±6.01	±0.98	1.19	6.01	0.98
sample 7	7	18.60	±0.99	±5.47	±0.55	0.99	5.47	0.55
sample 8	7	18.64	±0.91	±5	±0.4	0.91	5.00	0.40
sample 9	7	18.73	±1.01	±5.34	±0.6	1.01	5.34	0.60

(i) First instar larvae

Result - Sample integrity: Variation [InVar] based on selected Housekeeping Genes

print result pages

	n	BestKeeper	InVar. [± CP]	InVar. [± % CP]	InVar. [± x-fold]	InVar. [± CP]	InVar. [± % CP]	InVar. [± x-fold]
sample 1	7	19.49	±1.31	±6.44	±1.36	1.31	6.44	1.36
sample 2	7	19.77	±1.02	±4.72	±1.35	1.02	4.72	1.35
sample 3	7	19.55	±0.93	±4.59	±0.72	0.93	4.59	0.72
sample 4	7	19.89	±1.06	±5.28	±1.14	1.06	5.28	1.14
sample 5	7	20.18	±0.75	±3.82	±0.69	0.75	3.82	0.69
sample 6	7	20.04	±0.73	±3.67	±0.58	0.73	3.67	0.58
sample 7	7	19.59	±1.5	±7.24	±0.96	1.50	7.24	0.96
sample 8	7	19.52	±1.42	±7.04	±1.17	1.42	7.04	1.17
sample 9	7	19.57	±1.35	±6.77	±1.08	1.35	6.77	1.08

(j) Second instar larvae

Result - Sample integrity: Variation [InVar] based on selected Housekeeping Genes

print result pages

	n	BestKeeper	InVar. [± CP]	InVar. [± % CP]	InVar. [± x-fold]	InVar. [± CP]	InVar. [± % CP]	InVar. [± x-fold]
sample 1	7	20.46	±1.03	±4.89	±1.01	1.03	4.89	1.01
sample 2	7	20.69	±1.14	±5.43	±1.2	1.14	5.43	1.20
sample 3	7	20.54	±0.89	±4.49	±0.73	0.89	4.49	0.73
sample 4	7	20.63	±1.18	±5.64	±1.15	1.18	5.64	1.15
sample 5	7	20.68	±1	±4.93	±1.09	1.00	4.93	1.09
sample 6	7	20.50	±1.1	±5.33	±0.98	1.10	5.33	0.98
sample 7	7	19.97	±1.52	±7.04	±0.73	1.52	7.04	0.73
sample 8	7	20.02	±1.27	±5.83	±0.63	1.27	5.83	0.63
sample 9	7	19.98	±1.18	±5.32	±0.5	1.18	5.32	0.50

(k) Third instar larvae

Result - Sample integrity: Variation [InVar] based on selected Housekeeping Genes

print result pages

	n	BestKeeper	InVar. [± CP]	InVar. [± % CP]	InVar. [± x-fold]	InVar. [± CP]	InVar. [± % CP]	InVar. [± x-fold]
sample 1	7	20.69	±1.17	±5.35	±1.3	1.17	5.35	1.30
sample 2	7	21.25	±1.38	±6.56	±2.1	1.38	6.56	2.10
sample 3	7	21.09	±1.13	±5.23	±1.15	1.13	5.23	1.15
sample 4	7	20.71	±0.48	±2.34	±0.32	0.48	2.34	0.32
sample 5	7	20.49	±0.54	±2.83	±0.34	0.54	2.83	0.34
sample 6	7	20.57	±0.67	±3.45	±0.45	0.67	3.45	0.45
sample 7	7	20.26	±1.02	±4.7	±0.58	1.02	4.70	0.58
sample 8	7	20.78	±1.35	±6.44	±1.45	1.35	6.44	1.45
sample 9	7	20.87	±1.13	±5.4	±1.11	1.13	5.40	1.11

(l) Fourth instar larvae

Result - Sample integrity: Variation [InVar] based on selected Housekeeping Genes

print result pages

	n	BestKeeper	InVar. [± CP]	InVar. [± % CP]	InVar. [± x-fold]	InVar. [± CP]	InVar. [± % CP]	InVar. [± x-fold]
sample 1	7	21.90	±1.28	±5.63	±1.8	1.28	5.63	1.80
sample 2	7	21.87	±1.27	±5.57	±1.64	1.27	5.57	1.64
sample 3	7	21.89	±1.34	±5.85	±2.06	1.34	5.85	2.06
sample 4	7	21.25	±0.66	±3.17	±0.42	0.66	3.17	0.42
sample 5	7	21.04	±0.56	±2.64	±0.31	0.56	2.64	0.31
sample 6	7	21.18	±0.75	±3.5	±0.42	0.75	3.50	0.42
sample 7	7	21.52	±1.42	±6.36	±1.62	1.42	6.36	1.62
sample 8	7	21.37	±1.43	±6.41	±2.02	1.43	6.41	2.02
sample 9	7	21.37	±1.52	±7.05	±2.13	1.52	7.05	2.13

(m)Pupae

Result - Sample integrity: Variation [InVar] based on selected Housekeeping Genes

print result pages

	n	BestKeeper	InVar. [± CP]	InVar. [± % CP]	InVar. [± x-fold]	InVar. [± CP]	InVar. [± % CP]	InVar. [± x-fold]
sample 1	7	20.02	±0.71	±3.85	±0.68	0.71	3.85	0.68
sample 2	7	19.96	±0.53	±3.01	±0.51	0.53	3.01	0.51
sample 3	7	19.80	±0.56	±3.13	±0.49	0.56	3.13	0.49
sample 4	7	19.71	±0.39	±2.09	±0.24	0.39	2.09	0.24
sample 5	7	19.83	±0.22	±1.21	±0.16	0.22	1.21	0.16
sample 6	7	19.62	±0.43	±2.4	±0.27	0.43	2.40	0.27
sample 7	7	19.63	±0.41	±2.19	±0.23	0.41	2.19	0.23
sample 8	7	19.88	±0.25	±1.28	±0.19	0.25	1.28	0.19
sample 9	7	19.77	±0.32	±1.77	±0.22	0.32	1.77	0.22

(n) Adult Male

Result - Sample integrity: Variation [InVar] based on selected Housekeeping Genes

print result pages

	n	BestKeeper	InVar. [± CP]	InVar. [± % CP]	InVar. [± x-fold]	InVar. [± CP]	InVar. [± % CP]	InVar. [± x-fold]
sample 1	7	25.57	±0.63	±2.27	±0.88	0.63	2.27	0.88
sample 2	7	25.64	±0.56	±1.99	±0.8	0.56	1.99	0.80
sample 3	7	25.41	±0.48	±1.72	±0.55	0.48	1.72	0.55
sample 4	7	27.18	±0.8	±2.06	±3.03	0.80	2.06	3.03
sample 5	7	27.14	±0.84	±2.27	±3.14	0.84	2.27	3.14
sample 6	7	26.91	±0.91	±2.67	±2.98	0.91	2.67	2.98
sample 7	7	21.91	±1.08	±3.29	±0.12	1.08	3.29	0.12
sample 8	7	21.95	±1.09	±3.38	±0.13	1.09	3.38	0.13
sample 9	7	21.88	±1.07	±3.17	±0.12	1.07	3.17	0.12

(o) Adult Female

Result - Sample integrity: Variation [InVar] based on selected Housekeeping Genes

print result pages

	n	BestKeeper	InVar. [± CP]	InVar. [± % CP]	InVar. [± x-fold]	InVar. [± CP]	InVar. [± % CP]	InVar. [± x-fold]
sample 1	7	19.50	±0.53	±2.9	±0.17	0.53	2.90	0.17
sample 2	7	19.42	±0.64	±3.52	±0.19	0.64	3.52	0.19
sample 3	7	19.25	±0.57	±3.16	±0.16	0.57	3.16	0.16
sample 4	7	22.39	±0.71	±3.95	±1.67	0.71	3.95	1.67
sample 5	7	22.31	±0.64	±3.58	±1.59	0.64	3.58	1.59
sample 6	7	22.15	±0.73	±3.86	±1.53	0.73	3.86	1.53
sample 7	7	19.96	±0.43	±2.3	±0.23	0.43	2.30	0.23
sample 8	7	20.05	±0.47	±2.46	±0.26	0.47	2.46	0.26
sample 9	7	20.18	±0.36	±1.82	±0.2	0.36	1.82	0.20

(p) Adult Female, 6hPBM

Result - Sample integrity: Variation [InVar] based on selected Housekeeping Genes

print result pages

	n	BestKeeper	InVar. [± CP]	InVar. [± % CP]	InVar. [± x-fold]	InVar. [± CP]	InVar. [± % CP]	InVar. [± x-fold]
sample 1	7	21.75	±0.62	±3.02	±0.43	0.62	3.02	0.43
sample 2	7	21.85	±0.76	±3.58	±0.54	0.76	3.58	0.54
sample 3	7	21.80	±0.65	±3.31	±0.59	0.65	3.31	0.59
sample 4	7	21.55	±0.46	±2.09	±0.28	0.46	2.09	0.28
sample 5	7	21.56	±0.59	±2.68	±0.35	0.59	2.68	0.35
sample 6	7	21.82	±0.59	±2.96	±0.48	0.59	2.96	0.48
sample 7	7	21.69	±1.28	±6.2	±1.15	1.28	6.20	1.15
sample 8	7	21.72	±1.13	±5.36	±0.94	1.13	5.36	0.94
sample 9	7	21.96	±1.07	±5.05	±0.98	1.07	5.05	0.98

(q) *Aag2* cells

Result - Sample integrity: Variation [InVar] based on selected Housekeeping Genes

print result pages

	n	BestKeeper	InVar. [± CP]	InVar. [± % CP]	InVar. [± x-fold]	InVar. [± CP]	InVar. [± % CP]	InVar. [± x-fold]
sample 1	7	20.93	±0.8	±4.2	±1.48	0.80	4.20	1.48
sample 2	7	20.77	±0.88	±4.59	±1.44	0.88	4.59	1.44
sample 3	7	20.69	±0.73	±3.85	±1.04	0.73	3.85	1.04
sample 4	7	19.94	±1	±4.72	±1.44	1.00	4.72	1.44
sample 5	7	19.89	±1.05	±5.01	±1.49	1.05	5.01	1.49
sample 6	7	20.04	±1.08	±5.04	±2.03	1.08	5.04	2.03
sample 7	7	18.37	±0.81	±4.21	±0.22	0.81	4.21	0.22
sample 8	7	18.03	±0.76	±3.9	±0.19	0.76	3.90	0.19
sample 9	7	17.98	±0.89	±4.61	±0.24	0.89	4.61	0.24

Supplementary Table S2: BestKeeper descriptive statistic analysis

Dev. Stage	BestKeeper component	*ACT*	*eEF1α*	*α-tubulin*	*RPL8*	*RPL32*	*RPS17*	*GAPDH*
0-3h	std dev [± CP]	0.610	0.268	0.941	0.141	0.360	0.506	0.811
	BestKeeper vs. coeff. of corr. [r]	0.851	0.497	0.970	0.559	-0.727	-0.134	0.635
	p-value	0.004	0.172	0.001	0.118	0.027	0.729	0.067
3-6h	std dev [± CP]	0.579	0.826	0.292	0.487	0.499	0.583	0.247
	BestKeeper vs. coeff. of corr. [r]	0.624	0.752	-0.195	0.869	0.826	0.774	0.832
	p-value	0.073	0.019	0.613	0.002	0.006	0.014	0.005
6-9h	std dev [± CP]	0.636	0.635	1.327	0.340	0.341	0.534	1.148
	BestKeeper vs. coeff. of corr. [r]	0.943	0.985	0.948	0.844	-0.666	-0.921	0.917
	p-value	0.001	0.001	0.001	0.004	0.050	0.001	0.001
9-12h	std dev [± CP]	0.422	0.513	0.551	0.196	0.165	0.290	1.018
	BestKeeper vs. coeff. of corr. [r]	0.979	0.921	0.922	0.229	0.636	-0.805	0.892
	p-value	0.001	0.001	0.001	0.555	0.066	0.009	0.001
12-18h	std dev [± CP]	0.337	0.236	0.159	0.406	0.206	0.165	0.347
	BestKeeper vs. coeff. of corr. [r]	0.936	0.846	-0.424	0.991	0.820	0.735	0.948
	p-value	0.001	0.004	0.255	0.001	0.007	0.024	0.001
18-24h	std dev [± CP]	0.283	0.125	0.871	0.129	0.309	0.419	0.575
	BestKeeper vs. coeff. of corr. [r]	0.820	0.855	0.804	0.228	0.074	-0.310	0.589
	p-value	0.007	0.003	0.009	0.555	0.847	0.418	0.095
24-48h	std dev [± CP]	0.417	0.683	0.894	1.002	1.048	0.239	1.873
	BestKeeper vs. coeff. of corr. [r]	0.097	0.371	0.565	-0.424	-0.108	0.490	0.325
	p-value	0.802	0.324	0.113	0.255	0.780	0.180	0.393
48-72h	std dev [± CP]	1.161	1.444	0.668	1.031	0.378	0.862	1.219
	BestKeeper vs. coeff. of corr. [r]	0.684	0.675	0.369	0.593	0.689	0.972	-0.927
	p-value	0.042	0.046	0.329	0.092	0.040	0.001	0.001
1L	std dev [± CP]	0.986	0.971	0.514	0.478	1.027	0.481	1.710
	BestKeeper vs. coeff. of corr. [r]	0.815	0.104	0.451	-0.035	-0.585	0.891	0.330
	p-value	0.007	0.788	0.222	0.931	0.098	0.001	0.388
2L	std dev [± CP]	0.673	0.924	0.498	0.662	0.753	0.292	2.121
	BestKeeper vs. coeff. of corr. [r]	0.512	0.257	-0.271	-0.820	-0.017	0.564	0.968
	p-value	0.158	0.507	0.483	0.007	0.969	0.113	0.001

3L	std dev [± CP]	0.832	0.952	0.468	0.623	1.020	0.432	1.313
	BestKeeper vs. coeff. of corr. [r]	-0.200	0.833	0.535	0.648	-0.075	-0.499	0.616
	p-value	0.606	0.005	0.137	0.059	0.847	0.172	0.077
4L	std dev [± CP]	0.978	1.618	0.520	0.465	0.789	0.366	1.709
	BestKeeper vs. coeff. of corr. [r]	0.705	-0.131	0.859	0.577	-0.138	-0.803	0.795
	p-value	0.034	0.737	0.003	0.104	0.722	0.009	0.010
Pupae	std dev [± CP]	0.402	0.162	0.248	0.714	0.393	0.127	0.133
	BestKeeper vs. coeff. of corr. [r]	0.769	0.477	-0.396	0.772	-0.610	0.430	-0.357
	p-value	0.015	0.193	0.292	0.015	0.081	0.248	0.346
Adult Male	std dev [± CP]	1.509	2.133	2.339	2.701	1.416	0.932	3.059
	BestKeeper vs. coeff. of corr. [r]	0.996	0.999	0.988	0.994	0.999	0.994	0.996
	p-value	0.001	0.001	0.001	0.001	0.001	0.001	0.001
Adult Female	std dev [± CP]	0.972	1.568	1.254	1.693	1.043	0.931	0.406
	BestKeeper vs. coeff. of corr. [r]	0.987	0.951	0.994	0.995	0.962	0.977	0.918
	p-value	0.001	0.001	0.001	0.001	0.001	0.001	0.001
Adult Female PB	std dev [± CP]	0.152	0.372	0.896	0.998	0.501	0.991	0.697
	BestKeeper vs. coeff. of corr. [r]	0.777	0.156	0.330	0.252	-0.033	-0.036	0.313
	p-value	0.014	0.687	0.388	0.512	0.931	0.923	0.413
Aag2 Cells	std dev [± CP]	0.500	0.964	0.825	1.961	1.000	0.920	1.639
	BestKeeper vs. coeff. of corr. [r]	0.712	0.964	0.827	0.992	0.986	0.983	0.578
	p-value	0.032	0.001	0.006	0.001	0.001	0.001	0.104

[a]CP crossing point

[b]std dev standard deviation of the CP

(green) significantly correlated to *BestKeeper index*

(yellow) significantly correlated to *BestKeeper index* but with high standard deviation

(orange) gene with high standard deviation

Supplementary Table S3: Rankings from all three algorithms and resulting consensus. Consensus rankings are based on the geometric means of weightages in the form of stability values from geNorm and NormFinder, and a function of 1 -(*BestKeeper* vs. *Pearson* correlation coefficient value) from BestKeeper

Rank	Developmental stage											
	0-3h				3-6h				6-9h			
	BestKeeper	geNorm	NormFinder	Consensus	BestKeeper	geNorm	NormFinder	Consensus	BestKeeper	geNorm	NormFinder	Consensus
1	*α-tubulin*	*eEF1α/RPL8*	*RPL8*	*RPL8*	*RPL8*	*RPL8/RPS17*	*GAPDH*	*RPL8*	*eEF1α*	*ACT/eEF1α*	*ACT*	*eEF1α*
2	*ACT*		*eEF1α*	*eEF1α*	*GAPDH*		*RPL8*	*GAPDH*	*α-tubulin*		*eEF1α*	*ACT*
3	*RPL32*	*RPS17*	*ACT*	*ACT*	*RPL32*	*RPL32*	*RPL32*	*RPS17*	*ACT*	*RPL8*	*RPL8*	*RPL8*
4	*GAPDH*	*RPL32*	*RPS17*	*α-tubulin*	*RPS17*	*GAPDH*	*RPS17*	*RPL32*	*RPS17*	*GAPDH*	*GAPDH*	*GAPDH*
5	*RPL8*	*ACT*	*GAPDH*	*RPL32*	*eEF1α*	*α-tubulin*	*ACT*	*ACT*	*GAPDH*	*RPL32*	*RPL32*	*RPS17*
6	*eEF1α*	*GAPDH*	*RPL32*	*GAPDH*	*ACT*	*ACT*	*α-tubulin*	*eEF1α*	*RPL8*	*RPS17*	*RPS17*	*RPL32*
7	*RPS17*	*α-tubulin*	*α-tubulin*	*RPS17*	*α-tubulin*	*eEF1α*	*eEF1α*	*α-tubulin*	*RPL32*	*α-tubulin*	*α-tubulin*	*α-tubulin*
Rank	9-12h				12-18h				18-24h			
	BestKeeper	geNorm	NormFinder	Consensus	BestKeeper	geNorm	NormFinder	Consensus	BestKeeper	geNorm	NormFinder	Consensus
1	*ACT*	*ACT/eEF1α*	*ACT*	*ACT*	*RPL8*	*RPL8/GAPDH*	*eEF1α*	*RPL8*	*eEF1α*	*eEF1α/RPL8*	*eEF1α*	*eEF1α*
2	*α-tubulin*		*RPL32*	*eEF1α*	*GAPDH*		*ACT*	*GAPDH*	*ACT*		*ACT*	*ACT*
3	*eEF1α*	*α-tubulin*	*eEF1α*	*α-tubulin*	*ACT*	*ACT*	*RPL32*	*ACT*	*α-tubulin*	*RPL32*	*RPL8*	*RPL8*
4	*GAPDH*	*RPL32*	*α-tubulin*	*RPL32*	*eEF1α*	*eEF1α*	*RPS17*	*eEF1α*	*GAPDH*	*RPS17*	*RPL32*	*GAPDH*
5	*RPS17*	*RPL8*	*RPL8*	*RPL8*	*RPL32*	*RPL32*	*GAPDH*	*RPL32*	*RPS17*	*ACT*	*GAPDH*	*RPL32*
6	*RPL32*	*RPS17*	*RPS17*	*RPS17*	*RPS17*	*RPS17*	*RPL8*	*RPS17*	*RPL8*	*GAPDH*	*RPS17*	*α-tubulin*
7	*RPL8*	*GAPDH*	*GAPDH*	*GAPDH*	*α-tubulin*	*α-tubulin*	*α-tubulin*	*α-tubulin*	*RPL32*	*α-tubulin*	*α-tubulin*	*RPS17*
Rank	24-48h				48-72h				1L			
	BestKeeper	geNorm	NormFinder	Consensus	BestKeeper	geNorm	NormFinder	Consensus	BestKeeper	geNorm	NormFinder	Consensus
1	*α-tubulin*	*ACT/RPS17*	*ACT*	*ACT*	*RPS17*	*ACT/RPL8*	*RPL32*	*RPS17*	*RPS17*	*RPS17/α-tubulin*	*RPS17*	*RPS17*
2	*RPS17*		*RPS17*	*RPS17*	*GAPDH*		*RPS17*	*RPL32*	*ACT*		*α-tubulin*	*α-tubulin*
3	*RPL8*	*eEF1α*	*eEF1α*	*eEF1α*	*RPL32*	*RPS17*	*α-tubulin*	*ACT*	*RPL32*	*RPL8*	*RPL8*	*ACT*
4	*eEF1α*	*α-tubulin*	*α-tubulin*	*α-tubulin*	*ACT*	*RPL32*	*RPL8*	*RPL8*	*α-tubulin*	*ACT*	*ACT*	*RPL8*
5	*GAPDH*	*RPL32*	*RPL8*	*RPL8*	*eEF1α*	*α-tubulin*	*ACT*	*GAPDH*	*GAPDH*	*eEF1α*	*eEF1α*	*RPL32*

6	*RPL32*	*RPL8*	*RPL32*	*RPL32*	*RPL8*	*eEF1α*	*eEF1α*	*α-tubulin*	*eEF1α*	*RPL32*	*RPL32*	*eEF1α*
7	*ACT*	*GAPDH*	*GAPDH*	*GAPDH*	*α-tubulin*	*GAPDH*	*GAPDH*	*eEF1α*	*RPL8*	*GAPDH*	*GAPDH*	*GAPDH*
Rank	2L				3L				4L			
	BestKeeper	geNorm	NormFinder	Consensus	BestKeeper	geNorm	NormFinder	Consensus	BestKeeper	geNorm	NormFinder	Consensus
1	*GAPDH*	*RPS17/α-tubulin*	*RPS17*	*RPS17*	*eEF1α*	*RPS17/α-tubulin*	*α-tubulin*	*α-tubulin*	*α-tubulin*	*RPL8/α-tubulin*	*α-tubulin*	*α-tubulin*
2	*RPL8*		*ACT*	*RPL8*	*RPL8*		*RPL8*	*RPL8*	*RPS17*		*RPL8*	*RPL8*
3	*RPS17*	*RPL8*	*α-tubulin*	*GAPDH*	*GAPDH*	*ACT*	*RPS17*	*RPS17*	*GAPDH*	*ACT*	*RPS17*	*RPS17*
4	*ACT*	*ACT*	*RPL32*	*ACT*	*α-tubulin*	*RPL32*	*eEF1α*	*eEF1α*	*ACT*	*RPS17*	*ACT*	*ACT*
5	*α-tubulin*	*RPL32*	*eEF1α*	*α-tubulin*	*RPS17*	*RPL8*	*ACT*	*ACT*	*RPL8*	*RPL32*	*RPL32*	*GAPDH*
6	*eEF1α*	*eEF1α*	*RPL8*	*eEF1α*	*ACT*	*eEF1α*	*RPL32*	*GAPDH*	*RPL32*	*GAPDH*	*GAPDH*	*RPL32*
7	*RPL32*	*GAPDH*	*GAPDH*	*RPL32*	*RPL32*	*GAPDH*	*GAPDH*	*RPL32*	*eEF1α*	*eEF1α*	*eEF1α*	*eEF1α*
Rank	Pupae				Adult, Male				Adult, Female			
	BestKeeper	geNorm	NormFinder	Consensus	BestKeeper	geNorm	NormFinder	Consensus	BestKeeper	geNorm	NormFinder	Consensus
1	*RPL8*	*RPS17/eEF1α*	*RPS17*	*eEF1α*	*eEF1α*	*ACT/RPL32*	*eEF1α*	*RPL32*	*RPL8*	*RPL32/RPS17*	*ACT*	*ACT*
2	*ACT*		*eEF1α*	*RPS17*	*RPL32*		*ACT*	*eEF1α*	*α-tubulin*		*α-tubulin*	*α-tubulin*
3	*RPL32*	*GAPDH*	*GAPDH*	*GAPDH*	*ACT*	*eEF1α*	*RPL32*	*ACT*	*ACT*	*ACT*	*RPL32*	*RPS17*
4	*eEF1α*	*α-tubulin*	*α-tubulin*	*ACT*	*GAPDH*	*α-tubulin*	*α-tubulin*	*RPL8*	*RPS17*	*α-tubulin*	*RPS17*	*RPL32*
5	*RPS17*	*RPL32*	*ACT*	*α-tubulin*	*RPL8*	*RPL8*	*RPL8*	*GAPDH*	*RPL32*	*RPL8*	*RPL8*	*RPL8*
6	*α-tubulin*	*ACT*	*RPL32*	*RPL8*	*RPS17*	*GAPDH*	*GAPDH*	*α-tubulin*	*eEF1α*	*GAPDH*	*eEF1α*	*eEF1α*
7	*GAPDH*	*RPL8*	*RPL8*	*RPL32*	*α-tubulin*	*RPS17*	*RPS17*	*RPS17*	*GAPDH*	*eEF1α*	*GAPDH*	*GAPDH*
Rank	Adult, Female, 6h PBM				Aag2 Cells							
	BestKeeper	geNorm	NormFinder	Consensus	BestKeeper	geNorm	NormFinder	Consensus				
1	*ACT*	*α-tubulin/RPL8*	*ACT*	*ACT*	*RPL8*	*eEF1α/RPL32*	*eEF1α*	*RPL32*				
2	*α-tubulin*		*eEF1α*	*α-tubulin*	*RPL32*		*RPL32*	*eEF1α*				
3	*GAPDH*	*GAPDH*	*GAPDH*	*GAPDH*	*RPS17*	*RPS17*	*RPS17*	*RPS17*				
4	*RPL8*	*ACT*	*RPL32*	*RPL8*	*eEF1α*	*α-tubulin*	*α-tubulin*	*RPL8*				
5	*eEF1α*	*eEF1α*	*α-tubulin*	*eEF1α*	*α-tubulin*	*ACT*	*ACT*	*α-tubulin*				
6	*RPS17*	*RPL32*	*RPL8*	*RPL32*	*ACT*	*RPL8*	*RPL8*	*ACT*				
7	*RPL32*	*RPS17*	*RPS17*	*RPS17*	*GAPDH*	*GAPDH*	*GAPDH*	*GAPDH*				

Supplementary Table S4: *ctps* fold-change values as estimated with either single-normalizer genes, or different combinations of top-ranked genes, as denoted. $2^{-\Delta Ct_{ctps}}$ is the presumptive 'true' fold change.

	$2^{-\Delta\Delta Ct}$										
	ACT	*eEF1α*	*α-tubulin*	*RPL8*	*RPL32*	*RPS17*	*GAPDH*	top-two	top-three	*ACT/RPS17*	$2^{-\Delta Ct_{ctps}}$
0-3h	0.366	0.979	1.211	0.483	0.234	0.494	2.653	0.690	0.568	0.432	0.274
3-6h	0.272	0.269	1.366	0.295	0.231	0.195	0.461	0.343	0.281	0.227	1.667
6-9h	3.147	2.968	4.630	1.144	1.980	1.166	5.396	3.055	2.194	1.850	0.429
9-12h	2.076	1.598	2.248	1.214	1.145	0.640	0.220	1.814	1.940	1.108	1.375
12-18h	0.306	1.143	0.884	0.778	0.507	0.598	0.085	0.295	0.298	0.442	2.630
18-24h	0.772	1.285	0.441	0.011	0.743	0.822	0.440	1.010	1.004	0.800	2.798
24-48h	1.143	1.247	0.700	1.252	1.101	0.869	0.867	0.983	1.064	0.983	3.976
48-72h	1.182	1.120	0.544	1.556	1.665	1.510	0.491	1.585	1.459	1.360	3.892
1L	2.168	1.092	1.666	3.337	1.719	1.994	2.506	1.853	1.951	2.075	0.575
2L	2.420	2.088	1.144	4.799	2.583	2.598	4.609	2.507	3.008	2.507	0.897
3L	1.363	1.602	2.574	3.739	2.186	2.395	2.264	3.168	2.853	1.854	0.909
4L	1.896	0.856	4.299	3.089	1.251	1.541	2.343	3.585	2.624	1.690	0.208
Pupae	3.099	2.325	8.271	4.644	2.757	3.884	6.812	3.106	4.098	3.479	1.276
Adult, M	0.134	0.104	0.036	0.051	0.417	0.803	0.072	0.214	0.185	0.358	0.684
Adult, F	0.654	1.746	0.387	0.715	1.391	1.198	0.285	0.511	0.714	0.913	0.465
Adult, F, PBM	0.234	0.179	0.599	0.019	1.403	0.905	0.254	0.237	0.242	0.494	0.531
Aag2 Cells	1.673	1.611	0.407	0.465	0.792	0.421	1.847	1.126	0.805	0.803	0.483
